# Validation of ^125^I-hCG as a marker for elimination of hCG and stability of ^125^I-hCG after in vivo injection in humans

**DOI:** 10.1038/sj.bjc.6690566

**Published:** 1999-07

**Authors:** T B Christensen, J Marqversen, F Engbaek, P Berger, T Bacher, H von der Maase

**Affiliations:** Departments of Oncology, Clinical Physiology and Nuclear Medicine and Clinical Biochemistry, Aarhus University Hospital, Denmark; Department of Clinical Physiology and Nuclear Medicine, Herlev Hospital, University of Copenhagen, Denmark; Institute for Biomedical Aging Research, Austrian Academy of Sciences, Austria

**Keywords:** gonadotropins, human chorionic, radioiodinated hCG, in vivo elimination

## Abstract

We have recently introduced ^125^I-hCG as an elimination marker in patients with human chorionic gonadotrophin (hCG) producing testicular cancer. ^125^I-hCG is a well-known reagent in clinical biochemistry and is used extensively in hCG assays. Previous studies have shown that the iodination process leaves the hCG molecule mainly intact. The iodination, purification and stability of ^125^I-hCG tracer are described. The aim of the present study was to determine whether or not ^125^I is associated with hCG after the injection of ^125^I-hCG intravenously (i.v.) in humans. Three different methods were used. Following injection of ^125^I-hCG, the plasma disappearance of radioactivity and hCG were followed for a period of 28 days in 13 normal subjects. Serum from a normal healthy male following injection of ^125^I-hCG was analysed using a double antibody direct binding radioimmunoassay specific for holo-hCG and high performance liquid chromatography (HPLC). Following injection of ^125^I-hCG in eight normal healthy males and five normal healthy females, the disappearance of radioactivity and hCG showed identical paths in the 28 days follow-up period. The bindable radioactive fraction of immunologically active hCG in serum of a normal healthy male following injection of ^125^I-hCG was between 57.0% and 72.1%, and was constant over time. HPLC showed similar elution pattern of serum from a normal healthy male injected i.v. with ^125^I-hCG and ^125^I-hCG. Using three different methods, we were able concurrently to demonstrate the association of ^125^I with hCG in humans up to 28 days after injection of radiolabelled hCG i.v. Thus, information about the expected elimination of hCG can be obtained by following the elimination of activity in plasma after injection of ^125^I-hCG. © 1999 Cancer Research Campaign


*British Journal of Cancer* (1999) **80**(10), 1582–1587
© 1999 Cancer Research Campaign
Article no. bjoc.1999.0566

### Validation of $^{125}$I-hCG as a marker for elimination of hCG and stability of $^{125}$I-hCG after in vivo injection in humans

**TB Christensen[1,4], J Marqversen[2], F Engbaek[3], P Berger[5], T Bacher[2] and H von der Maase[1]**

Departments of [1]Oncology, [2]Clinical Physiology and Nuclear Medicine, and [3]Clinical Biochemistry, Aarhus University Hospital, Denmark; [4]Department of Clinical Physiology and Nuclear Medicine, Herlev Hospital, University of Copenhagen, Denmark; [5]Institute for Biomedical Aging Research, Austrian Academy of Sciences, Austria

**Summary** We have recently introduced $^{125}$I-hCG as an elimination marker in patients with human chorionic gonadotrophin (hCG) producing testicular cancer. $^{125}$I-hCG is a well-known reagent in clinical biochemistry and is used extensively in hCG assays. Previous studies have shown that the iodination process leaves the hCG molecule mainly intact. The iodination, purification and stability of $^{125}$I-hCG tracer are described. The aim of the present study was to determine whether or not $^{125}$I is associated with hCG after the injection of $^{125}$I-hCG intravenously (i.v.) in humans. Three different methods were used. Following injection of $^{125}$I-hCG, the plasma disappearance of radioactivity and hCG were followed for a period of 28 days in 13 normal subjects. Serum from a normal healthy male following injection of $^{125}$I-hCG was analysed using a double antibody direct binding radioimmunoassay specific for holo-hCG and high performance liquid chromatography (HPLC). Following injection of $^{125}$I-hCG in eight normal healthy males and five normal healthy females, the disappearance of radioactivity and hCG showed identical paths in the 28 days follow-up period. The bindable radioactive fraction of immunologically active hCG in serum of a normal healthy male following injection of $^{125}$I-hCG was between 57.0% and 72.1%, and was constant over time. HPLC showed similar elution pattern of serum from a normal healthy male injected i.v. with $^{125}$I-hCG and $^{125}$I-hCG. Using three different methods, we were able concurrently to demonstrate the association of $^{125}$I with hCG in humans up to 28 days after injection of radiolabelled hCG i.v. Thus, information about the expected elimination of hCG can be obtained by following the elimination of activity in plasma after injection of $^{125}$I-hCG.

**Keywords**: gonadotropins; human chorionic; radioiodinated hCG; in vivo elimination

Repeated measurements of human chorionic gonadotrophin (hCG) are used to monitor the effect of treatment in patients with hCG-producing testicular cancer. Following chemotherapy, the disappearance of hCG from serum is assumed to follow first-order kinetics with a fixed half-life of fewer than 3 days (Bosl et al, 1981; Toner et al, 1990; Motzer et al, 1992, 1993; Bosl and Chaganti, 1994; Bosl and Head, 1994; Murphy et al, 1994). As factors like changes in volume of distribution, treatment-induced organ toxicity, weight loss and repeated blood transfusions could influence the disappearance of hCG, we assumed the expected monoexponential decline to be a too simple model. In order to study the hCG elimination during chemotherapy, we recently introduced $^{125}$I-hCG as an elimination marker and found that the disappearance of $^{125}$I-hCG and immunologically measured hCG was quite different from the commonly used monoexponential model with a half-life of less than 3 days (Christensen et al, 1999).

After injection of 3 MBq $^{125}$I-hCG intravenously (i.v.), serum samples were drawn at defined time intervals and analysed for hCG content using an immunofluorometric assay. Radioactivity for the same samples was measured in parallel in a $\gamma$-counter. Serum disappearance curves for $^{125}$I-hCG and hCG were plotted. If the disappearance curves showed a similar pattern, we interpreted the slow disappearance as caused by a slow elimination rather than a production of hCG from vital tumour cells.

$^{125}$I-hCG is a well-known reagent in clinical biochemistry and is used extensively in hCG assays. Previous studies have shown that the iodination process leaves the hCG molecule mainly intact (Catt et al, 1972). During iodination, two epitopes on the $\alpha$ chain are destroyed (Berger et al, 1988). The structural integrity of hCG and its ability to bind to specific antibodies and LH/hCG receptors is well retained after iodination. After bolus injection of $^{125}$I-hCG and hCG into rats, no difference in disappearance was seen (Markkanen et al, 1980).

The present investigations were initiated to answer the following questions of importance for the utilization of $^{125}$I-hCG as an elimination marker:

Does iodination of hCG affect its elimination in humans?
Does the drop of radioactivity in serum reflect elimination of $^{125}$I-hCG, metabolites, or dissociation of $^{125}$I from injected $^{125}$I-hCG i.v.?

### MATERIALS AND METHODS

The study was approved by the local ethics committee and all volunteers gave their informed written consent before entering the study.

### Iodination of hCG

The hCG used for labelling was supplied by LEO Pharmaceutical Co., Denmark. The crude hCG was obtained from the urine of

*Received 26 June 1998*
*Revised 19 February 1999*
*Accepted 7 April 1999*

*Correspondence to:* TB Christensen, Department of Oncology, Aarhus University Hospital, DK-8000, Aarhus C, Denmark pregnant women and delivered without additives. LEO used the crude hCG as raw material for their human ovary luteinizing hormone (LH)-stimulating pharmaceutical Physex®. The hCG was purified to 3000 IU $mg^{-1}$, sterilized, tested for pyrogens, oestrogens and abnormal toxicity, and conformed according to (Council of Europe, 1986). The labelling of the hCG was carried out by Isopharma A/S, Kjeller, Norway. The following procedure was used: for labelling 1.5 ml hCG (1 mg $ml^{-1}$) were dissolved in 0.01 M phosphate-buffered saline (PBS), pH 7.4, mixed with 90 MBq $Na^{125}I$ in a tube containing 60 μg iodogen®. The mixture was allowed to incubate for 10 min before it was transferred to an empty tube. For purification, the reaction mixture was then applied to a PD-10 column (Pharmacia, Sephadex G-25, medium coarse), equilibrated with 1 mg human serum albumin (HSA) $ml^{-1}$ PBS and eluted with 1 mg HSA $ml^{-1}$ PBS. Following the first 2.5 ml, eight fractions of 0.5 ml each were collected. Fractions with less than 2% iodide were collected, mixed and diluted to 2 MBq $ml^{-1}$ with 1 mg HSA $ml^{-1}$ PBS. The purification procedure was carried out twice. The product was sterile filtered using a 0.22 μm filter (Durapore), tested for radiochemical purity, using high-voltage-electrophoresis on the day of production, and for pH, pyogens and bacterial growth 14 days after production. The $^{125}$I-hCG used had a labelling per cent of > 88%, radiochemical purity with < 1.7% iodide, no detectable contamination with $^{125}$I-albumin, specific activity 50–60 MBq $mg^{-1}$, pH 7.0–7.5, and was sterile and free of pyogens (LAL < 175 EU 20 $ml^{-1}$).

High-voltage-electrophoresis was used to test the radiochemical stability of $^{125}$I-hCG following incubation at 4°C for 5, 7, 14, 20 and 29 days respectively. The iodide content was < 5%. To investigate the influence of the iodination process on the integrity of hCG, one sample of hCG was taken through the same procedure for labelling *except* for the iodogen and iodination step. The product was then analysed for intact hCG and free β-hCG following 18 days of storage using immunofluorometric assays. In the labelled and unlabelled product, intact hCG concentrations of 42 000 IU $l^{-1}$ and 56 000 IU $l^{-1}$ and free β-hCG concentrations of 270 IU $l^{-1}$ and 550 IU $l^{-1}$, respectively, were found. Differences in concentrations are caused by difference in the dilution step.

### Dosimetry

After injection of 2.66 MBq of $^{125}$I-hCG in a healthy normal male, serum samples were obtained once a day for 21 days. The plasma disappearance curve of activity from $^{125}$I counted in the spectrum of 15–75 KeV was constructed. The data were fitted a triexponential model minimizing least squares using non-linear regression according to the Marquardt–Levenberg method. Area under curve was calculated. Assuming the total activity was deposited in the patient, a volume of distribution of 7.3l, the total accumulated radiation dose was calculated to be < 0.05 mSv following injection of 3 MBq $^{125}$I-hCG.

### *Elimination of $^{125}$I-hCG in normal healthy subjects*

Giving 400 mg KI orally at least 1 h before injection of $^{125}$I-hCG prevented thyroid uptake of $^{125}$I. A well-functioning i.v. catheter was placed in the cubital vein of the subject. Serum samples were drawn prior to injection of $^{125}$I-hCG to determine background activity. Then, 3 MBq $^{125}$I-hCG (Isopharma A/S, Kjeller, Norway) were given as a single i.v. bolus injection. The specific activity of the labelled hCG was 50 MBq $mg^{-1}$ and the concentration was 3000 IU $mg^{-1}$. Thus, the quantity of hCG given was 180 IU. The elimination of hCG was studied in two ways:

(a.) Immunologically by measuring the plasma disappearance of the total hCG, using a modified Wallac immunofluorometric assay. The assay was modified by doubling the sample volume and changing detecting antibody to INN-hFSH-158 specific for the α-chain epitope $\alpha_5$. The capturing antibody used was INN-hCG-2 specific for the β-chain epitope $\beta_5$ (Dirnhofer et al, 1994). The sensitivity was 0.1 IU $l^{-1}$ (3.IS) and LH cross-reactivity 0.4%.

(b.) By measuring the rate of plasma disappearance of activity of $^{125}$I. Radioactivity was measured in a well counter (COBRA auto gamma, Packard, USA) set to detect within the energy range 15–75 KeV. The background activity has been subtracted from all the presented data. All plasma samples were counted at the same time to avoid calculated correction for the decay of $^{125}$I. The relative counting error was less than 5%. Serum samples were taken twice a week for the first 2 weeks and then once a week for the following weeks. The $^{125}$I concentrations are presented as counts $min^{-1}$ for a 3 ml aliquot of plasma.

### *Immunoadsorption*

Serum samples drawn from a normal healthy volunteer (male, age 36 years, weight 86 kg, height 187 cm) after i.v. injection of 2.97 MBq $^{125}$I-hCG at 0 h, 2 h, 4 h, 1, 2, 3, 4 days were analysed using a double antibody direct binding radioimmunoassay. Monoclonal antibodies (mAb) against the β subunit of hCG (code: INN-hCG 22) were incubated with 0.4 ml of serum overnight at 4°C. To determine non-specific binding (NSB), 0.4 ml of serum were incubated with mAb and excess (100 IU) of non-labelled hCG (Pregnyl®, Organon, The Netherlands). After addition of 0.2 ml immunoadsorbent consisting of goat Ig-antimouse Ig covalently linked to sepharose 6B (Pharmacia, Uppsala, Sweden) and incubation for 3 h at room temperature with vigorous agitation, activity was counted in a γ-counter (Wallac, Turku, Finland) for 15 min to determine the total activity ($T_a$) for each sample. The tubes were then washed three times using PBS–Tween-80 and counted for 15 min to determine total bound activity ($B_t$) and NSB. Counter background activity (BG) was determined with empty tubes. As more than 90% of radioactivity after iodination of hCG is associated with the α-subunit of hCG, this assay will measure holo-hCG (Markkanen et al, 1980).

All samples were run in duplicate. Mean values were used for calculation.

Total added activity corrected for background activity ($T_{acorr}$) was calculated as $T_{acorr} = T_a\text{-BG}$ and specifically bound activity as $B_s = B_t\text{-NSB}$.

Per cent $B_s$ of $T_{acorr}$ was calculated as $\%B_s = \dfrac{B_s*100}{T_{acor}}$ %

### *High-performance liquid chromatography*

Serum samples drawn from a normal healthy volunteer (male, age 40 years, weight 75 kg, height 181 cm) at 2 h, 4 h, 1, 2, 3 and 4 days after injection of 1.75 MBq $^{125}$I-hCG were analysed by high-performance liquid chromatography (HPLC) using a Waters Deltapac C18 column equilibrated with 0.1% TFA (trifluoracetic acid) in water, and flow 1 ml $min^{-1}$. From each serum sample, five samples of 100 μl each of serum diluted to 50% by sterile saline (0.9% sodium chloride in water) to prevent clotting of the system,

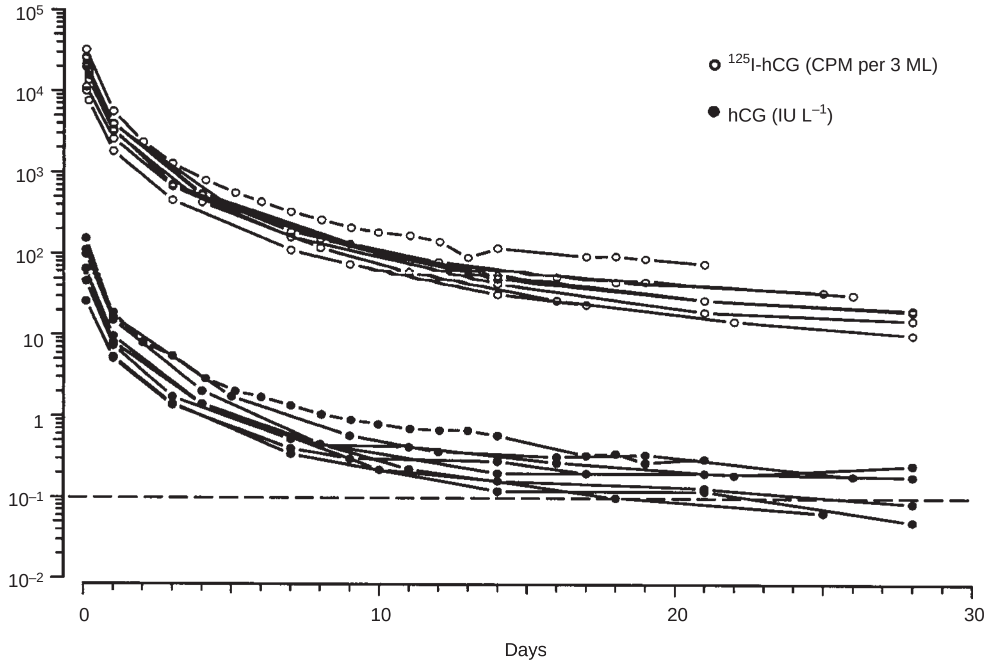

**Figure 1** Plasma disappearance of radioactivity and hCG following injection of $^{125}$I-hCG in eight normal healthy males

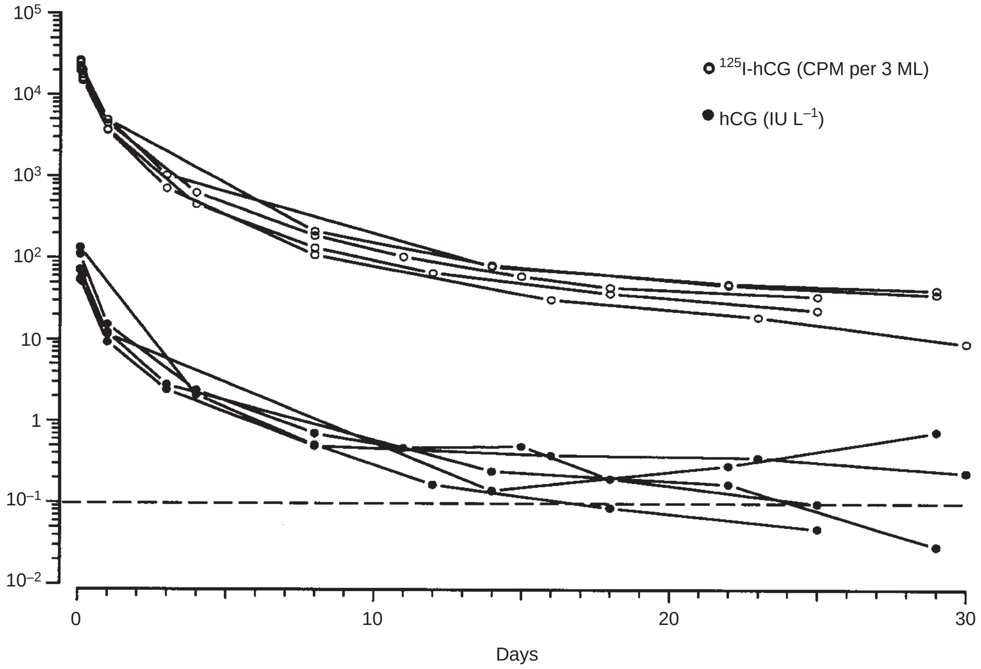

**Figure 2** Plasma disappearance of radioactivity and hCG following injection of $^{125}$I-hCG in five normal healthy females were applicated to the column consecutively. After the fifth application, the chromatography was continued as reverse phase (RP) chromatography by increasing the concentration of (acetonitrile + 0.1% TFA) from 0% at 5 min to 100% at 20 min. From the 25th min the column was again eluted with 0.1% TFA.

The outflow from the column was collected in 3-min fractions in order to determine the low radioactivity. This procedure, unfortunately, loses some resolution in detection of elution time but this was necessary due to the low count rate which was undetectable by the HPLC-radioactivity flow detector. The first six fractions were collected during application of the first four samples. The remaining ten fractions were collected during the RP period. The fractions were counted for radioactivity in the energy range 15–75 KeV covering the energy range of $^{125}$I for 10 min each using a γ-well counter (Packard). Samples were analysed in order of increasing activity to avoid carry over between samples in the column. Counter background activity was determined on empty tubes and subtracted from each sample.

*© 1999 Cancer Research Campaign*

**Table 1**

| Sample time | $T_{acorr}$ | $B_t$ | NSB | $B_s$ | $\%B_s$ of $T_{acorr}$ |
|---|---|---|---|---|---|
| 2t | 52 500 | 39 884 | 3499 | 36 385 | 69.3% |
| 4t | 36 340 | 27 331 | 2714 | 24 617 | 67.7% |
| 1d | 11 245 | 8726 | 1049 | 7677 | 68.3% |
| 2d | 4841 | 3975 | 725 | 3250 | 67.1% |
| 3d | 3146 | 2882 | 612 | 2270 | 72.2% |
| 4d | 2056 | 1772 | 598 | 1172 | 57.0% |

Values for $T_{acorr}$, $B_t$, NSB, and $B_s$ are given in counts per 15 min.

Analysing unlabelled hCG and $^{125}$I-hCG used for i.v. injection, UV detector (280 nm) and radioactivity detector (15–75 KeV) were used.

### RESULTS

### The in vivo elimination of $^{125}$I-hCG in normal healthy subjects

Eight healthy males with a median age of 37 years (range 29–45), median weight of 78 kg (range 66.4–83.0) and a median height of 185 cm (range 171–195) were injected with a median dose of 2.92 MBq $^{125}$I-hCG (range 2.48–3.10). Plasma disappearance of hCG and radioactivity are shown in Figure 1.

Five healthy females with a median age of 41 years (range 37–42), median weight of 60.0 kg (range 49.2–62.8) and a median height of 166 cm (range 163–168) were injected with a median dose of 2.89 MBq $^{125}$I-hCG (range 2.63–3.02). Plasma disappearance of hCG and radioactivity are shown in Figure 2.

The plasma disappearance of radioactivity and hCG showed identical paths from injection of $^{125}$I-hCG to day 28 in both males and females. The plasma disappearance showed an initial fast elimination phase followed by a phase with a decreasing rate of disappearance over time. In order to describe the covariation between $^{125}$I-hCG and hCG, measurements were logarithmically transformed and a linear least squares regression analysis was applied followed by a *t*-test procedure for significance. An intercept $\alpha = 5.53$ ($P < 0.0001$), slope $\beta = 1.045$ ($P < 0.0001$) and correlation coefficient between $^{125}$I-hCG and hCG, $r^2 = 0.94$ ($P < 0.0001$) were found.

### Immunoadsorption

The radioactivity counted on 3 ml of proband serum *before* injection of $^{125}$I-hCG was identical to background activity. The percentage of immunoreactive tracer in the serum of a healthy proband injected with a bolus of $^{125}$I-hCG remained constant over time between 67.1 and 72.1% of total radioactivity (Table 1). Only after 4 days did it apparently drop to 57%, but this might be due to the vulnerability of the test system at extremely low count rates.

### HPLC

The radioactivity counted on 3 ml of proband serum *before* injection of $^{125}$I-hCG was identical to background activity. No radioactivity was detected in the first eight fractions, indicating no free iodine. The elution pattern of radioactivity in serum samples drawn from 2 h to 3 days after injection of $^{125}$I-hCG is identical to the elution pattern of $^{125}$I-hCG (Figure 3). The radioactivity of $^{125}$I-hCG eluted in fractions 13 and 14. The radioactivity in serum after injection of $^{125}$I-hCG eluated in fraction 13 and 14 constant over time. In Figure 4, the percentage of radioactivity applied to the column from each sample is shown. The recovery was 101% at 2 h, 92% at 4 h, 91% at day 1, 118% at day 2 and 69% at day 3.

HPLC of $^{125}$I-hCG used for injection showed a UV 280 nm and radioactivity detector signal at the same elution time. HPLC of the

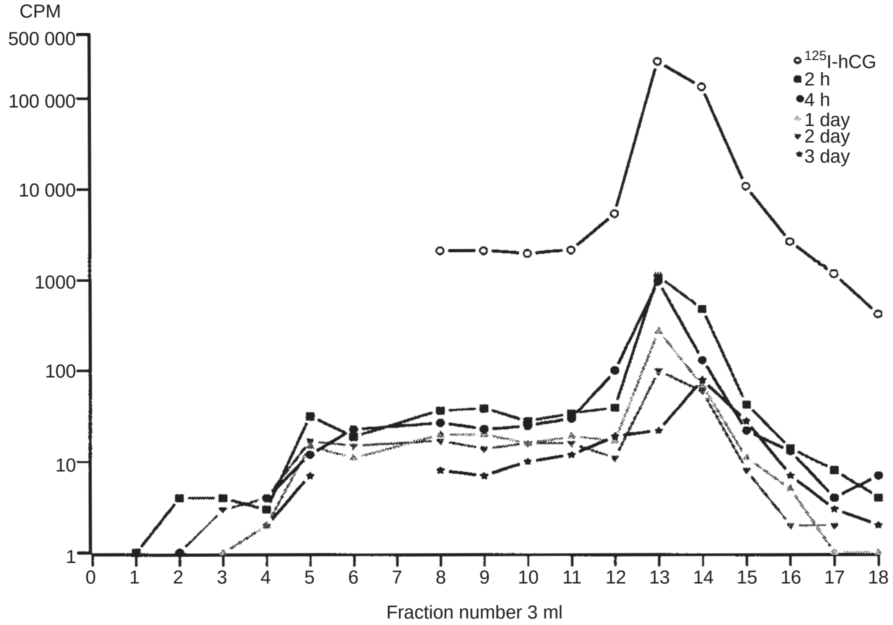

**Figure 3** HPLC of $^{125}$I-hCG and serum from normal healthy male following injection of $^{125}$I-hCG. Values given as counts per minute (CPM) in each fraction

*© 1999 Cancer Research Campaign* 

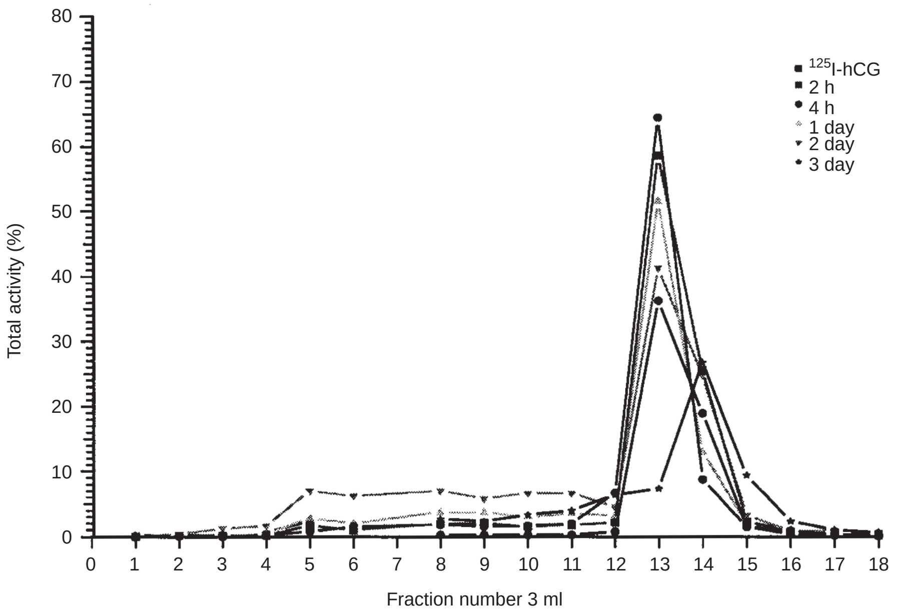

**Figure 4** HPLC of $^{125}$I-hCG and serum from normal healthy male following injection of $^{125}$I-hCG. Values given as percentages of total applied activity

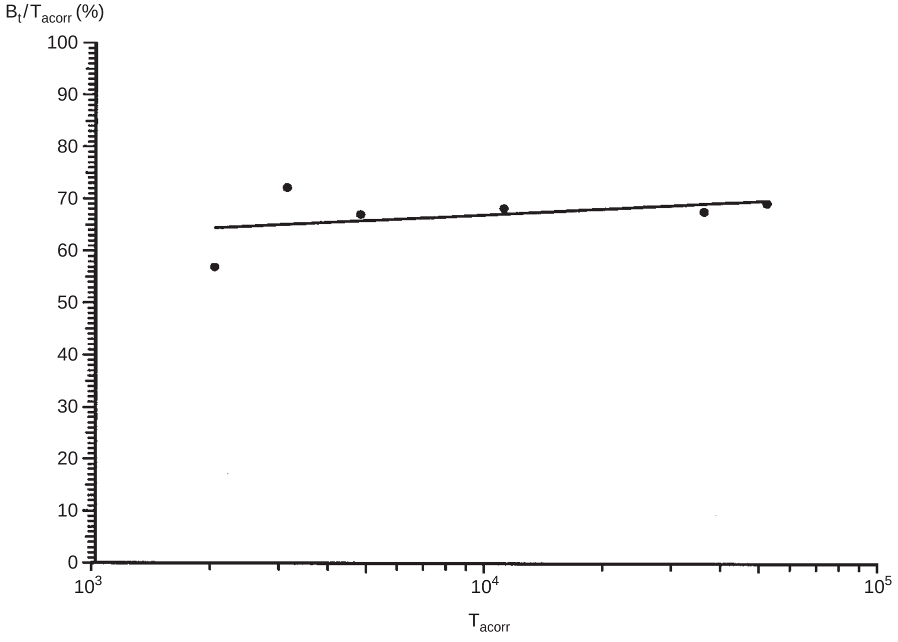

**Figure 5** $BtT_{acorr}$ (%) as a function of $T_{acorr*}$ Linear regression $y = 1.66*\ln(x) + 51.7$. SE on slope is ± 1.77. The value 1.66 found is not significantly different from 0 (*t*-test)

unlabelled hCG used as raw material for $^{125}$I-hCG showed a UV 280 nm detector signal at the same time as radioactivity detector signal during HPLC of $^{125}$I-hCG.

### DISCUSSION

We have recently introduced $^{125}$I-hCG as an elimination marker in patients with hCG producing testicular cancer (Christensen et al, 1999). For this purpose, it is important that $^{125}$I is associated with hCG in vivo. Following injection of $^{125}$I-hCG in eight healthy males and five healthy females, the plasma disappearance of radioactivity ($^{125}$I-hCG) and immunologically measured hCG showed identical paths from the day of injection to 28 days after injection (Figures 1 and 2). The correlation between $^{125}$I-hCG and hCG measurements was found to be statistically significant ($r^2$ = 0.9443, $P$ < 0.0001). The multiphasic shape of the plasma disappearance of $^{125}$I-hCG and hCG clearly demonstrates the inappropriateness of calculating one half-life for the elimination of hCG, as the half-life steadily increases over the 28-day observation period in this study. Serum hCG values in the eight healthy males were less than 0.25 IU $l^{-1}$ and in the five healthy females less than 0.5 IU $l^{-1}$ before injection of $^{125}$I-hCG. Thus, the main part of immunologically measured hCG originates from the

© 1999 Cancer Research Campaign injected $^{125}$I-hCG. No side-effects were seen following the injection of $^{125}$I-hCG. The crude hCG was delivered without additives. Following iodination we were not able, with the present methods, to find any difference in immunological reactivity or elution pattern of labelled, unlabelled, or crude hCG material, suggesting that the hormone remains intact following iodination using the described iodogen and purification method. Furthermore, we analysed LH concentrations in serum samples from some normal healthy males and females after injection of $^{125}$I-hCG and were able to detect a small transient decrease in LH concentrations (data not presented) suggesting that the labelled hormone was still hormonally active.

After iodination of hCG, immunoreactivity of $^{125}$I-hCG is maximum 70–80% of the total radioactivity. These percentages are only found when highly purified hCG (14 000 IU mg$^{-1}$) is used for labelling purposes. We used material purified from urine of pregnant women with an immunological activity of 3000 IU mg$^{-1}$. Using double-antibody direct-binding radioimmunassay, we were able to bind a constant fraction of immunologically active hCG in serum following injection of $^{125}$I-hCG. The bindable fractions were between 57 and 72% (Table 1, Figure 5). This is comparable to the known immunoreactivity achievable after iodination of hCG. At day 4, we were only able to bind 57%, but this could be due to the vulnerability of the test system at low count rates. The bindable fraction of $^{125}$I-hCG was 49%. This is probably due to a content of damaged hCG molecules following iodination and storage (radiation damage). Iodination of hCG destroys two out of 22 epitopes on the hCG molecule as described by Berger et al (1988). Clearance of radioactive iodide, following ingestion of KI, damaged hCG molecules, β-hCG and α-hCG produced from urine of pregnant women, is very rapid from the circulation of man (Wehmann and Nisula, 1980; Liu et al, 1989). Thus, shortly after injection of $^{125}$I-hCG, the main part of radioactivity in serum will be associated with hCG, as damaged fractions of protein will be cleared from the circulation.

HPLC on serum from a healthy normal male following i.v. injection of $^{125}$I-hCG showed no radioactivity in the first eight fractions, indicating no free iodine. The radioactivity constantly eluted in fractions 13 and 14 (Figures 3 and 4). HPLC of $^{125}$I-hCG also showed the radioactivity to elute in fractions 13 and 14. Thus, $^{125}$I-hCG and radioactivity in serum after injection of $^{125}$I-hCG showed an identical elution pattern. As HPLC of crude hCG and $^{125}$I-hCG showed the same elution times, $^{125}$I was associated with hCG in the $^{125}$I-hCG used for injection.

In summary, we were able to demonstrate concurrently in each method the association of $^{125}$I with hCG in humans for up to 28 days after i.v. injection of radiolabelled hCG by use of three different methods. Thus, $^{125}$I-hCG is stable in vivo and information of the expected elimination of hCG can be obtained by following the elimination of activity in plasma after injection of $^{125}$I-hCG.

### ACKNOWLEDGEMENTS

This study has been supported with grants from: Danish Cancer Society, Grosserer Valdemar Foersom og hustru Thyra Foersom, født Otto's fond, Clinical Research Unit Aarhus University Hospital, Clinical Research Unit Herlev University Hospital of Copenhagen, Ingeniør August Frederik Wedell Erichens Legat, Direktør Jacob Madsens og Hustru Olga Madsens Fond, and Hans und Blanca Moser Stiftung.

### REFERENCES

Berger P, Panmoung W, Khaschabi D, Mayregger B and Wick G (1988) Antigenic features of human follicle stimulating hormone delineated by monoclonal antibodies and construction of an immunoradiometric assay. *Endocrinology* **123**: 2351–2359

Bosl GJ and Chaganti RSK (1994) The use of tumor markers in germ cell malignancies. *Hematol Oncol Clin North Am* **8**: 573–587

Bosl GJ, Lange PH, Nochomovitz LE, Goldmann A, Fraley EE, Rosai J, Johnson K and Kennedy BJ (1981) Tumor markers in advanced nonseminomatous testicular cancer. *Cancer* **47**: 572–576

Bosl GJ and Head MD (1994) Serum tumor marker half-life during chemotherapy in patients with germ cell tumors. *Int J Biol Markers* **9**: 25–28

Catt KJ, Dufau ML and Tsuruhara T (1972) Radioligand-receptor assay of luteinizing hormone and chorionic gonadotropin. *J Clin Endocrinol Metab* **34**: 123–132

Christensen TB, Engbaek F, Marqversen J, Nielsen SL, Kamby C and von der Maase H (1999) $^{125}$I-hCG as an elimination marker in the evaluation of hCG decline during chemotherapy in patients with testicular cancer. *Br J Cancer* **80**: 1577–1581

Council of Europe (1986) European Pharmacopoeia, 2nd Edn.

Dirnhofer S, Madersbacher S, Bidart JM, Ten Kortenaar PB, Spottl G, Mann K, Wick G and Berger P (1994) The molecular basis for epitopes on the free beta-subunit of human chorionic gonadotrophin (hCG), its carboxyl-terminal peptide and the hCG beta-core fragment. *J Endocrinol* **141**: 153–162

Liu L, Southers JL, Cassels JW Jr, Banks SM, Wehmann RE, Blithe DL, Chen HC and Nisula BC (1989) Structure-kinetic relationships of choriogonadotropin and related molecules. *Am J Physiol* **256**: E721–E724

Markkanen S, Tollikko K, Jaaskelainen K and Rajaniemi H (1980) Effect of radioiodination on the structural integrity, receptor and antibody-binding activity and circulatory behavior of human choriogonadotropin. *Horm Res* **12**: 32–45

Motzer RJ, Gulati SC, Crown JP, Weisen S, Doherty M, Herr H, Fair W, Sheinfeld J, Sogani P, Russo P, Bosl GJ (1992) High-dose chemotherapy and autologous bone marrow rescue for patients with refractory germ cell tumors. Early intervention is better tolerated. *Cancer* **69**: 550–556

Motzer RJ, Mazumdar M, Gulati SC, Bajorin DF, Lyn P, Valamis V and Bosl GJ (1993) Phase II trial of high-dose carboplatin and etoposide with autologous bone marrow transplantation in first-line therapy for patients with poor-risk germ cell tumors. *J Natl Cancer Inst* **85**: 1828–1835

Murphy BA, Motzer RJ, Mazumdar M, Vlamis V, Nisselbaum J, Bajorin D and Bosl GJ (1994) Serum tumor marker decline is an early predictor of treatment outcome in germ cell tumor patients treated with cisplatin and ifosfamide salvage chemotherapy. *Cancer* **73**: 2520–2526

Toner GC, Geller NL, Tan C, Nisselbaum J and Bosl GJ (1990) Serum tumor marker half-life during chemotherapy allows early prediction of complete response and survival in nonseminomatous germ cell tumors. *Cancer Res* **50**: 5904–5910

Wehmann RE and Nisula BC (1980) Renal clearance rates of the subunits of human chorionic gonadotropin in man. *J Clin Endocrinol Metab* **50**: 674–679

© 1999 Cancer Research Campaign
